# Metabolite Profiling in a Diet-Induced Obesity Mouse Model and Individuals with Diabetes: A Combined Mass Spectrometry and Proton Nuclear Magnetic Resonance Spectroscopy Study

**DOI:** 10.3390/metabo13070874

**Published:** 2023-07-23

**Authors:** João P. P. Vieira, Filip Ottosson, Amra Jujic, Vladimir Denisov, Martin Magnusson, Olle Melander, João M. N. Duarte

**Affiliations:** 1 Department of Experimental Medical Science, Faculty of Medicine, Lund University, 22184 Lund, Sweden; joao.vieira@med.lu.se; 2 Wallenberg Centre for Molecular Medicine, Lund University, 22100 Lund, Sweden; amra.jujic@med.lu.se (A.J.); martin.magnusson@med.lu.se (M.M.); 3 Department of Clinical Sciences-Malmö, Faculty of Medicine, Lund University, 20502 Malmö, Sweden; filip.ottosson@med.lu.se (F.O.); olle.melander@med.lu.se (O.M.); 4 Department of Cardiology, Skåne University Hospital, 21428 Malmö, Sweden; 5 Biomedical Engineering Division, Department of Clinical Sciences-Lund, Faculty of Medicine, Lund University, 22100 Lund, Sweden; vladimir.denisov@med.lu.se; 6 Hypertension in Africa Research Team, North-West University, Potchefstroom 2520, South Africa

**Keywords:** metabolomics, LC-MS, NMR, plasma, biomarkers

## Abstract

Mass spectrometry (MS) and nuclear magnetic resonance (NMR) spectroscopy techniques have been used extensively for metabolite profiling. Although combining these two analytical modalities has the potential of enhancing metabolite coverage, such studies are sparse. In this study we test the hypothesis that combining the metabolic information obtained using liquid chromatography (LC) MS and ^1^H NMR spectroscopy improves the discrimination of metabolic disease development. We induced metabolic syndrome in male mice using a high-fat diet (HFD) exposure and performed LC-MS and NMR spectroscopy on plasma samples collected after 1 and 8 weeks of dietary intervention. In an orthogonal projection to latent structures (OPLS) analysis, we observed that combining MS and NMR was stronger than each analytical method alone at determining effects of both HFD feeding and time-on-diet. We then tested our metabolomics approach on plasma from 56 individuals from the Malmö Diet and Cancer Study (MDCS) cohort. All metabolic pathways impacted by HFD feeding in mice were confirmed to be affected by diabetes in the MDCS cohort, and most prominent HFD-induced metabolite concentration changes in mice were also associated with metabolic syndrome parameters in humans. The main drivers of metabolic disease discrimination emanating from the present study included plasma levels of xanthine, hippurate, 2-hydroxyisovalerate, S-adenosylhomocysteine and dimethylguanidino valeric acid. In conclusion, our combined NMR-MS approach provided a snapshot of metabolic imbalances in humans and a mouse model, which was improved over employment of each analytical method alone.

## 1. Introduction

Obesity is associated with increased risk of developing cardio-metabolic pathology, including fatty liver disease, type 2 diabetes mellitus (T2D), hypertension, myocardial infarction, stroke, musculoskeletal disease, depression, dementia and various types of cancers [1]. Metabolomics affords the measurement of many metabolites, the abundance of which in biological specimens is a proximal reporter of disease and/or therapeutics [2,3]. Hence, the use of metabolomics tools to profile metabolic shifts in obesity could provide insight into metabolite signatures characteristic of obesity [4]. Metabolic shifts induced by obesity across the various organs are reflected in the plasma metabolome [5,6].

Mass spectrometry (MS) and proton nuclear magnetic resonance (^1^H-NMR) spectroscopy constitute the primary analytical technologies used in metabolomics-based studies [7]. MS is a highly sensitive method for the detection of metabolites in biological samples (concentrations 10–100 nmol/L), although signal detection is affected by the nature of the experimental conditions and instrumental settings, thus requiring quantification through the employment of numerous metabolite standards [7,8]. On the other hand, despite its lower sensitivity (>1 μmol/L) and selectivity (peak overlapping), the employment of NMR spectroscopy for metabolomics studies offers the advantage of relatively simple sample processing, non-destructive character, high reproducibility [7,9] and predictable proportionality between signal and the concentration of nuclei within molecules of the sample [7,10]. Besides the quantitative analysis of metabolites, NMR spectra can be used as a fingerprint of metabolite concentrations that report the systemic metabolic status.

We aimed at testing the power of combining ^1^H-NMR spectroscopy and MS for determining plasma metabolome alterations in a diet-induced obesity mouse model that develops metabolic syndrome [11]. After having developed the NMR-MS combined approach, we tested the metabolome analysis on diabetic and non-diabetic individuals recruited for the Malmo Diet and Cancer Study [12].

## 2. Materials and Methods

### 2.1. Animals

**Figure 1:**
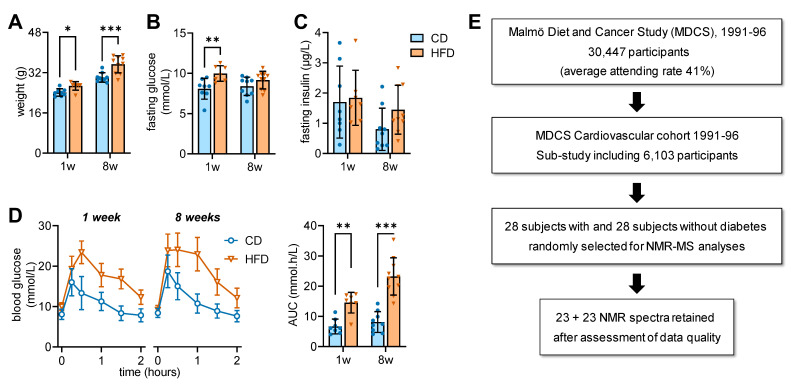
Metabolic characteristics of CD and HFD-fed mice (**A**–**D**), and MDCS subject recruitment and sample selection (**E**). Body weight was larger for HFD than CD-fed mice (**A**). Blood glucose concentrations (**B**) were increased in HFD-fed mice for 1 week. Plasma insulin concentrations (**C**) were increased in HFD-fed animals for 8 weeks. Glucose clearance in a glucose tolerance test (GTT) was reduced by HFD feeding for 1 and 8 weeks, as evidenced by both increased areas under the curve (AUC) of the GTT (**D**). Data is shown as mean ± SD. Bar graphs are overlaid onto individual data points, with triangles and circles indicating CD and HFD mice, respectively. Asterisks above data-points indicate significant differences relative to CD-fed mice as indicated (* *p* < 0.05, ** *p* < 0.01, *** *p* < 0.001), based on Fischer’s LSD post hoc comparison after significant effects ANOVA.

Experiments were performed according to EU Directive 2010/63/EU, approved by the Malmö/Lund Committee for Animal Experiment Ethics (5123/2021), and are reported following the ARRIVE guidelines (Animal Research: Reporting In Vivo Experiments, NC3Rs initiative, UK). C57BL/6J mice (8 weeks old) were purchased from Taconic Biosciences (Köln, Germany) and housed in groups of 2–5 animals on a 12 h light-dark cycle with lights on at 07:00, room temperature of 21–23 °C and humidity at 55–60%. Because of the important sex dimorphism that we observed in diet-induced obesity in mice (e.g., [13,14,15]), this study used only males. After habituating to the facility for 1 week, mice were randomly assigned to being fed either a high-fat diet (HFD) containing 60% kcal of saturated fat with a total energy of 5.21 kcal/g (D12492, Research Diets, New Brunswick, NJ, USA) or a composition-matched control diet (CD) containing 10% kcal of saturated fat with a total energy of 3.82 kcal/g (D12450J, Research Diets) for a duration of 1 or 8 weeks [15]. Gross food consumption was measured by weighing the amount of food added weekly and that remaining uneaten. Caloric intake tended to be higher in mice fed the HFD. Namely, caloric intake in kcal/mouse/day (cage average, n = 2) was 9.3 ± 1.0 for CD and 11.5 ± 2.3 for HFD at 1 week, and 9.8 ± 0.1 for CD and 10.7 ± 1.0 for HFD across 8 weeks. Severity of metabolic syndrome was evaluated with a glucose tolerance test (GTT) conducted as previously detailed [14]. In brief, after 6 h fasting, blood from the *vena saphena* was collected to determine plasma insulin using ELISA (#10-1247-10, Mercodia, Uppsala, Sweden), and tail-tip glucose was measured before and 2 h after intraperitoneal administration of glucose (2 g/kg). Metabolic characteristics of the mice (Figure 1A–D) were in general agreement with those in previous studies [11,15]. More specifically, HFD-fed mice in this study developed obesity with glucose intolerance, but not hyperinsulinaemia, when compared to CD-fed mice.

### 2.2. MDCS Cohort Description

All participants provided written informed consent and the study was approved by the Ethics Committee of Lund University, Lund, Sweden (LU 51-90). Between the years 1991 and 1996, a prospective, population-based study, “The Malmo Diet and Cancer Study” (MDCS), was conducted in the city of Malmö, Sweden, which has been previously described [12]. The study included anthropometrical measurements, blood sample donations and questionnaires at the baseline examination (n = 30,447). All subjects born between 1926–1945 and living in Malmö were invited to participate. In order to study cardiovascular risk factors and early atherosclerosis by collecting data on anthropometry, blood samples and ultrasound of the right carotid artery, a sample of the study population (n = 6103) was randomized into a sub study, “The Malmö Diet and Cancer Cardiovascular Cohort”. MS metabolomics has been conducted on this population (e.g., [5,6]). Citrated plasma from the baseline examination (1991–1996) was available. A total of 28 subjects with and 28 subjects without prevalent diabetes at the baseline examination were randomly selected for this combined NMR-MS exploratory study. Four samples were only used for MS analysis and not available for NMR spectroscopy. Samples from 6 subjects were excluded from NMR analyses after quality control assessment, resulting in a total sample size of 23 individuals in each group (Figure 1E, Table 1).

### 2.3. ^1^H-NMR

Mice were sacrificed under brief isoflurane anaesthesia, trunk blood was collected into heparinized tubes and plasma was stored at −80 °C. Mouse and human plasma metabolites were extracted as previously described [15]. Briefly, plasma and methanol were mixed at 1:3, vortexed and sonicated on ice for 30 min. After centrifugation at 13,000× *g* and 4 °C for 30 min, supernatants were dried in a Savant SpeedVac (Thermofisher Scientific, Göteborg, Sweden) concentrator operating at room temperature. Dried samples were dissolved in 600 μL of deuterium oxide in 100 mmol/L sodium phosphate buffer, pH 7.4, with 0.01% NaN_3_. Sodium fumarate (0.3 µmol) was added as internal standard, and samples were transferred into 5 mm Wilmad NMR tubes (Sigma-Aldrich, Taufkirchen, Germany). For mouse plasma, ^1^H-NMR spectra were acquired on an 11.7 T Varian VNMRS spectrometer equipped with a 5 mm PFG-TRP probe (Agilent technologies, Santa Clara, CA, USA) and using a Carr–Purcell–Meiboom–Gill sequence with water suppression, spectral width of 8 kHz, acquisition time of 2 s, relaxation delay of 10 s and 520 acquisitions. For human plasma, ^1^H-NMR spectra were acquired on a Bruker Avance III HD 14.1 T spectrometer equipped with a standard TCI cryoprobe using the ZGPR pre-saturation pulse sequence for water suppression with spectral width of 9 kHz, 3 s acquisition time and a relaxation delay of 22 s. The number of acquisitions varied according to initial plasma volume to attain similar signal-to-noise ratios (ranging from 194–522).

NMR spectra were processed in MATLAB 2021b (MathWorks, Natick, MA USA) using the General NMR Analysis Toolbox (GNAT) [16]. After alignment of the fumarate peak to 6.5 ppm and phase adjustment, the real part of the spectra (spectral points between 0 and 12 ppm) was saved for analysis. After aligning spectra using fumarate as reference, we removed spectral points corresponding to chemical shifts of residual water (mouse: 4.65–5.16 ppm; human: 4.55–5.16 ppm), methanol (3.32–3.35 ppm), fumarate reference peak (6.45–6.58 ppm) and citrate (only in human plasma: 2.47–2.68 ppm). Spectral points were normalized to the total spectral area. Peaks were identified by detection of local maxima, and these spectral points were used for subsequent analysis. Retained data points were then thresholded at 2-fold the noise level measured between 0 and 0.2 ppm. Any chemical shift with more than 50% of spectral points below the threshold was discarded.

Spectral points were assigned to metabolites with Chenomx NMR Suite 9.0 (Chenomx, Edmonton, AB, Canada) and previous publications [8,10,17,18,19,20] (Supplementary Figures S1 and S2).

### 2.4. Mass Spectrometry (MS)

Plasma metabolites were extracted with the addition of six volumes of a methanol:water 4:1 (*v*:*v*) mixture containing stable isotope-labelled internal standards from Cambridge Isotope Laboratories (Andover, MA, USA), as in previous studies [5,6]. After 30 min of incubation, the samples were centrifuged for 20 min at 14,000× *g* and the supernatants were transferred to glass vials. Extracted samples were separated on an Acquity UHPLC BEH Amide column (1.7 μm, 2.1 mm × 100 mm, Water Corporation, Milford, MA, USA) prior to MS analyses in a UHPLC-QTOF-MS System (Agilent 1290 LC, 6550 MS). Solvent A consisted of H_2_O, with 10 mmol/L ammonium formate and 0.1% formic acid. Solvent B consisted of acetonitrile with 0.1% formic acid. Gradient separation was performed as follows: 0–3 min, 100–95% B; 3–6 min, 95–80% B; 6–13 min, 80–70% B; 13–14 min, 70–40% B; 14–16 min, 40%; 16–17 min, 100% B. The flow rate was 0.4 mL/min and the sample injection volume was 2 µL. The auto-sampler was kept at 16 °C. Mass spectrometry was performed in positive electrospray ionization. The sheath gas temperature was set at 350 °C and the sheath gas flow at 12 L/min. The drying gas flow was 14 L/min and was delivered at 200 °C. The mass spectra were acquired at a rate of 1 spectrum/s and the mass range was set to 50–1000 *m*/*z*. MS/MS data was acquired at 20 eV and an isolation width of 1.3 *m*/*z*. Pooled plasma samples were used as quality controls and injected after every five analytical samples and run at the beginning of each batch, in order to capture analytical drift and condition the column to maintain high repeatability. Quality control samples were used to calculate the technical variation of the measurements.

Metabolites were annotated either by correct matching of MS/MS fragmentation and *m*/*z* with The Human Metabolome Database (HMDB) [21] and METLIN [22] (level 2 annotation), or by matching chromatographic retention time, *m*/*z* and MS/MS fragmentation with in-house metabolite library, consisting of synthetic standards (level 1 annotation). Level 1 and level 2 annotations correspond to annotation confidence as defined by the Metabolomics Standards Initiative [23]. Information about all annotated metabolites is included in Supplementary Table S1.

Data pre-processing was performed using the Find by Formula (FbF) algorithm in Mass Hunter Profinder B.06.00 (Agilent). FbF uses lists of known molecular formulae and retention times to extract metabolite spectra from mass spectrometry raw data. Allowed ion adducts included (M+H)^+^ and (M+NH_4_)^+^. Using low-order, nonlinear, locally estimated smoothing functions to create correction curves, metabolites’ intensities were normalized based on the measurement of metabolites in quality control samples [24].

### 2.5. Data Analysis

Z-scores of processed NMR spectral points and MS metabolite concentrations were analysed in SIMCA v.17.0.2 (Umetrics, Umeå, Sweden) via automated orthogonal projection to latent structures (OPLS) regression for diet and time-on-diet (mice), or for diabetes prevalence (humans). Cross-validation was used to infer on the predictive power of each OPLS model. OPLS regression models were validated using permutation tests (100 permutations for each Y variable). Variable importance in projection (VIP) scores greater than 1 were considered important for the projection of the OPLS regression model. OPLS model quality was assessed using the cumulative R^2^ (fraction of Y variation modelled) and cumulative Q^2^ (fraction of Y variation predicted by the model) obtained from SIMCA.

Variables with VIP > 1 were used for pathway analysis of NMR alone, MS alone and combined NMR-MS data in MetaboAnalyst 5.0 [25], using both the Kyoto Encyclopedia of Genes and Genomes (KEGG) and the Small Molecule Pathway Database (SMPDB) as pathway libraries [26,27]. When multiple NMR spectral points were available, that with the highest VIP score was used for pathway analysis. MetaboAnalyst computes a P-value for the difference of each metabolic pathway (a group of functional-associated metabolites), rather than each metabolite alone, and employs a pathway topology analysis to determine the pathway impact score, which represents the importance of a given pathway in relation to the global metabolic network. False-discovery rate (FDR) was used to compute adjusted P-values for false positives in the pathway analysis. Outcomes from all MetaboAnalyst analyses are provided in supplementary documents S1 and S2).

ANOVA for diet, time and their interaction for the characterization of mouse metabolic phenotype, Student t-tests, Mann–Whitney tests and Pearson correlation analyses were performed in Prism 9.3.0 (GraphPad, San Diego, CA, USA). Given the exploratory nature of the MDCS analysis, Pearson correlations were not corrected for multiple testing.

## 3. Results

### 3.1. Plasma Metabolomics in HFD-Fed Mice

**Figure 2:**
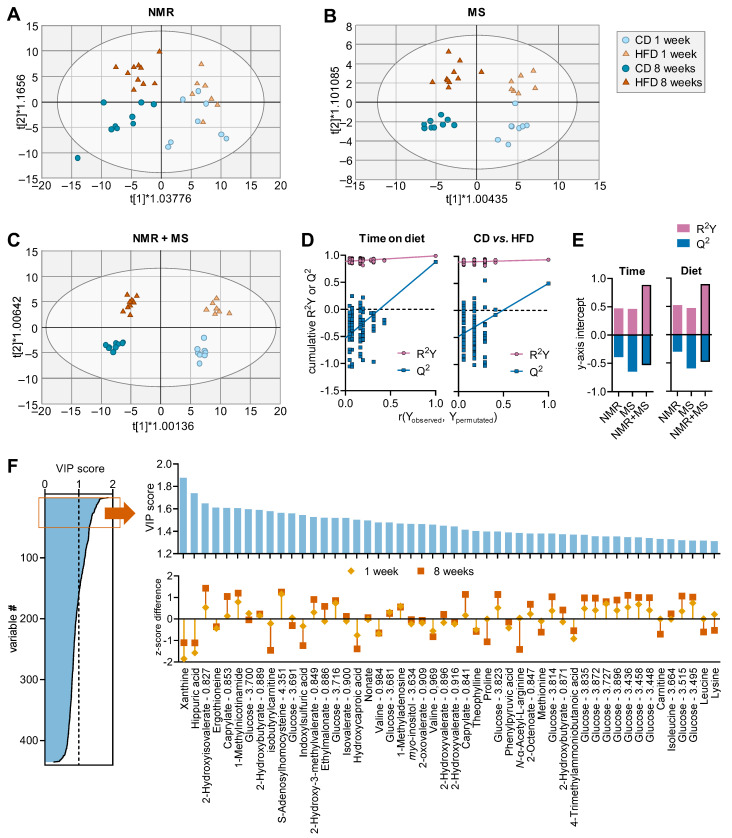
Multivariate data analysis using OPLS regression on samples from HFD and CD-fed mice. Plots showing group separation in the OPLS score space for analysis of ^1^H-NMR spectroscopy data (**A**), metabolite concentrations from MS (**B**) and their combination (**C**). Ellipses in score plots (**A**–**C**) represent Hotelling’s T2 (95%). (**D**) Typical response permutation test plot (n = 100) for the OPLS model in (**C**). (**E**) R^2^ and Q^2^ values corresponding to *y*-axis intercepts are represented for OPLS models in (**A**–**C**). (**F**) VIP scores estimated from OPLS model in (**C**) for all MS-determined metabolites and NMR spectral points, and expansion of the top 50 variables with highest VIP score, that is, the 50 strongest discriminating variables. Calculated z-score difference between HFD and CD at either 1 and 8 weeks is shown along the respective VIP score, with positive and negative values indicating HFD-induced increase and decrease concentration, respectively. Variables with z-score differences close to zero are likely associated with temporal changes (time-on-diet).

The OPLS regression applied to NMR data generated a model composed of two predictive components and one orthogonal component with cumulative R^2^ = 0.712 and Q^2^ = 0.432 (Figure 2A). OPLS model validation via permutation analysis resulted in R^2^ = 0.470 and Q^2^ = −0.389 for time-on-diet, and R^2^ = 0.524 and Q^2^ = −0.302 for diet effect.

OPLS regression of MS-measured metabolites generated a model composed of two predictive components and two orthogonal components with cumulative R^2^ = 0.899 and Q^2^ = 0.815 (Figure 2B). Model validation via permutation analysis revealed R^2^ = 0.461 and Q^2^ = −0.647 for time-on-diet, and R^2^ = 0.471 and Q^2^ = −0.598 for diet effect.

OPLS regression applied to NMR and MS data together resulted in improved separations of the four experimental groups using a model composed of two predictive and five orthogonal components with R^2^ = 0.964 and Q^2^ = 0.687 (Figure 2C). Model validation via permutation analysis resulted in R^2^ = 0.885 and Q^2^ = −0.564 for time-on-diet, and R^2^ = 0.896 and Q^2^ = −0.420 for diet effect (Figure 2D), which represent an improved outcome when compared to each dataset analysed alone (Figure 2E).

Altogether, we identified 160 NMR and MS variables with a VIP score above 1 (Figure 2F). From these, some of the top scoring metabolites included (metabolites from NMR with ppm in parenthesis): xanthine, hippuric acid, 2-hydroxyisovalerate (2-HIV; 0.827 ppm), ergothioneine, caprylate (0.853 ppm), 1-methylnicotinamide, glucose (3.700 ppm), 2-hydroxybutyrate (0.889 ppm), isobutyrylcarnitine, S-adenosylhomocysteine (4.351 ppm), indoxylsulfuric acid, 2-hydroxy-3-methylvalerate and/or caprate (0.849 ppm), ethylmalonate (0.886 ppm), isovalerate (0.900 ppm) and hydroxycaproic acid (Figure 2F). Not surprisingly, HFD-induced metabolic alterations were larger after 8 weeks than after 1 week of treatment, as inferred from z-score differences between HFD and CD (Figure 2; see also Supplementary Figure S6).

**Figure 3:**
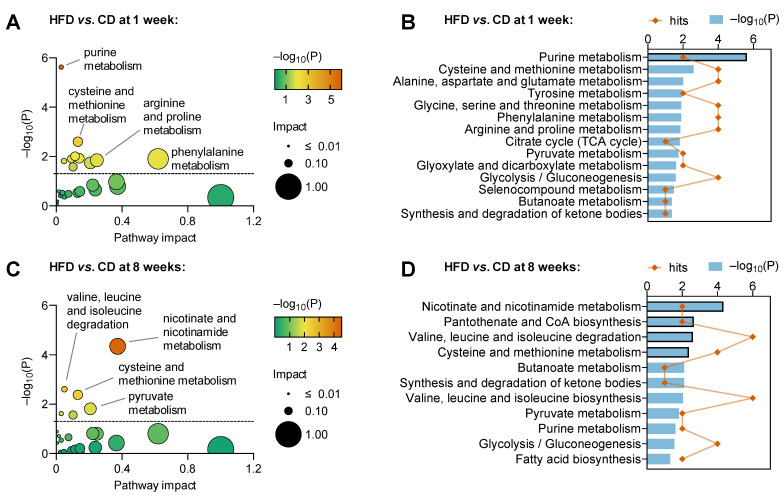
Metabolic pathway analysis after 1 week (**A**,**B**) and 8 weeks (**C**,**D**) of HFD intervention. MetaboAnalyst with the KEGG database was used to analyse z-scores from metabolites with VIP > 1 in OPLS regression for combined MS and NMR spectroscopy data. Panels (**A**,**C**) show the pathway impact determined using MetaboAnalyst and respective significance (*p*-values). Graphs in panels (**B**,**D**) include significant findings, that is pathways with unadjusted *p* < 0.05. Orange symbols and blue bars represent number of hits (metabolites analysed in that pathway) and −log_10_(P), respectively. Bars highlighted with black border indicate pathways in which significance survives false discovery rate (FDR) correction. Pathways with impact score = 0 are not represented in (**A**,**C**) but are listed in (**B**,**D**). Dashed lines in panels (**A**,**C**) indicate *p* = 0.05.

Group average z-scores from metabolites with VIP > 1 in the OPLS regression resulting from NMR and MS combined were then used for pathway analysis in MetaboAnalyst (Supplementary Document S1). For this, we used both the KEGG database (Figure 3) and the SMPDB database (Supplementary Figure S1). Purine metabolism appeared strongly affected in mice exposed to HFD for 1 week, relative to CD. Pathways of amino acid metabolism and cellular fuelling (that is, energy metabolism) also appear to be impacted by HFD (Figure 3A). However, only purine metabolism is significant after correction for multiple analyses (Figure 3B). After 8 weeks of HFD feeding, diet had further effects on other pathways, and purine metabolism became less prominently affected. Namely, HFD feeding for 8 weeks significantly impacted nicotinate and nicotinamide metabolism, pantothenate and coenzyme-A biosynthesis, the degradation of valine, leucine and isoleucine and the metabolism of cysteine and methionine (Figure 3C,D; Supplementary Figure S3). Notably, the combination of MS+NMR data for metabolic pathway analysis allowed the discovery of more pathways than using data from either technique in separate (Supplementary Figure S4).

### 3.2. Plasma Metabolomics in Individuals with Type 2 Diabetes

**Figure 4:**
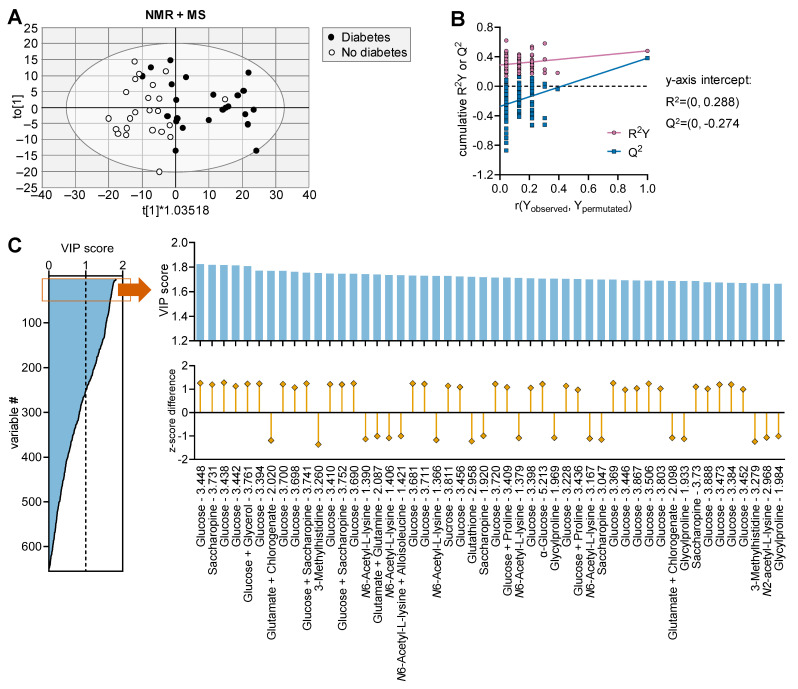
Multivariate data analysis using OPLS regression on samples from individuals with and without diabetes. (**A**) Plot showing group separation in the OPLS score space for the combined analysis of ^1^H-NMR spectroscopy data and metabolite concentrations from MS. (**B**) Response permutation test plot (n = 100) for the OPLS model in (**A**). (**C**) VIP scores estimated from OPLS model in panel (**A**) for all MS-determined metabolites and NMR spectral points, and expansion of the top 50 variables with highest VIP score, that is, the 50 strongest discriminating variables, along with calculated z-score difference between individuals with and without diabetes. Ellipse in score plot (**A**) represents Hotelling’s T2 (95%).

OPLS regression of NMR and MS data from individuals in the MDCS was composed of one predictive component and six orthogonal components with cumulative R^2^ = 0.479 and Q^2^ = 0.381. Plasma samples of diabetic and non-diabetic subjects are partially discriminated within the OPLS scoring space (Figure 4A). The model validation via permutation analysis performed for the response variable (diabetes versus no diabetes) resulted in R^2^ = 0.288 and Q^2^ = −0.274 (Figure 4B). We obtained 252 NMR and MS variables with VIP > 1, which included many chemical shifts corresponding to glucose signals in NMR spectroscopy (Figure 4C). Besides glucose, the metabolites with the highest VIP scores were from NMR spectroscopy and included (chemical shift in parenthesis): saccharopine (3.731 ppm), glutamate + chlorogenate (2.020 ppm), glucose + saccharopine (3.741 ppm), 3-methylhistidine (3.260 ppm), *N*6-acetyl-L-lysine (1.390 ppm), glutamate + glutamine (2.087 ppm), *N*6-acetyl-L-lysine + Alloisoleucine (1.421 ppm), sucrose (3.811 ppm), glutathione (2.958 ppm), glycylproline (1.969 ppm), *N*-α-acetyl-L-lysine (2.968 ppm), glutamate (2.041 ppm), 2-oxoisocaproate (2.070 ppm), lysine (1.883 ppm), *N*-acetylcysteine (2.049 ppm), isoleucine (1.497 ppm), arginine (1.657 ppm), 3-hydroxyisobutyrate (1.057 ppm), 4-hydroxybutyrate (3.578 ppm), butyrate (1.547 ppm), glycocholate (1.064 ppm), NADPH (4.049 ppm), methionine (2.153 ppm), 3,5-dibromotyrosine (2.872 ppm), 2-oxocaproate (1.560 ppm), 5-hydroxylysine (1.550 ppm), lysine + arginine (1.725 ppm), UMP (3.970 ppm), glutamine (2.141 ppm), alloisoleucine (1.336 ppm) and dimethylglycine (2.859 ppm).

**Figure 5:**
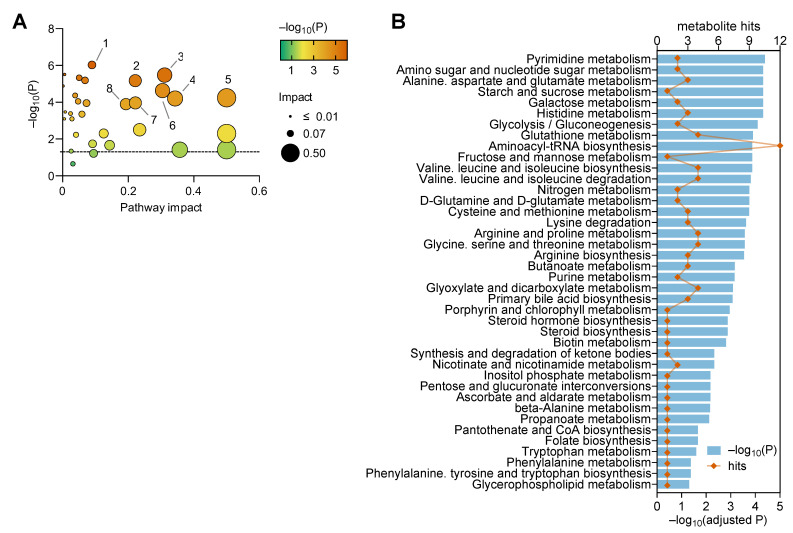
Metabolic pathway analysis depicting the impact of diabetes on systemic metabolism. MetaboAnalyst with the KEGG database was used to analyse z-scores from metabolites with VIP > 1 in OPLS regression for combined MS and NMR spectroscopy data. (**A**) Pathway impact determined using MetaboAnalyst and respective significance (*p*-values). (**B**) Significant findings after false discovery rate adjustment (adjusted *p* < 0.05; bars) and respective number of hits (metabolites analysed in that pathway; symbols). Pathways with impact score = 0 are not represented in (**A**) but are listed in (**B**). Dashed line in panel A indicates *p* = 0.05, and labels are as follows: 1—pyrimidine metabolism; 2—histidine metabolism; 3—alanine, aspartate and glutamate metabolism; 4—cysteine and methionine metabolism; 5—glutamine and glutamate metabolism; 6—glutathione metabolism; 7—arginine and proline metabolism; 8—arginine biosynthesis.

Pathway analysis in MetaboAnalyst (Supplementary Document S2) revealed that T2D affects 40 out of the 43 metabolic pathways in the KEGG database (Figure 5), which include all pathways identified in as significantly changing upon HFD feeding in mice. We obtained similar findings when using the SMPDB database (Supplementary Figure S4).

**Figure 6:**
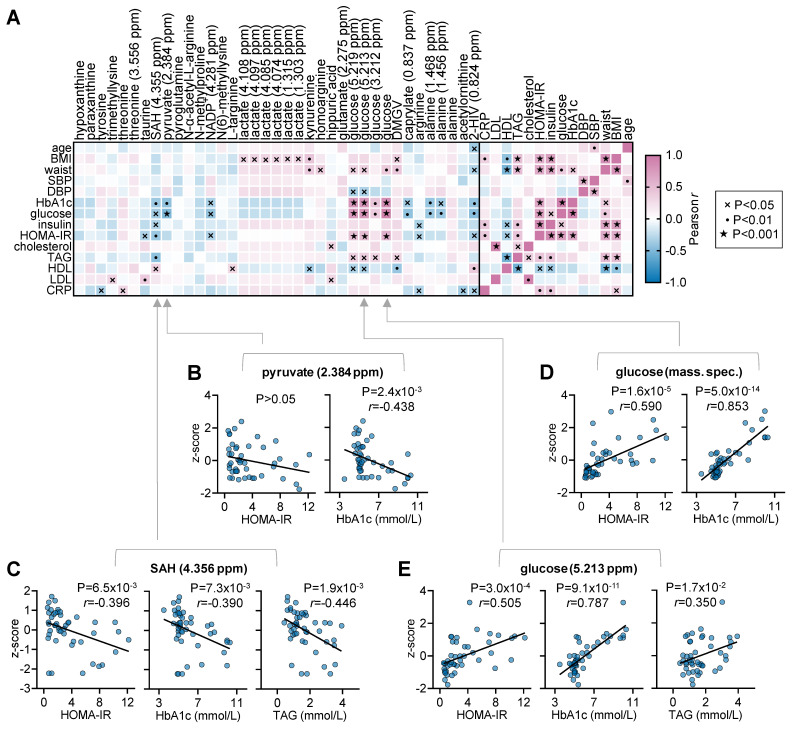
Cross-sectional analysis of selected variables in plasma from human subjects with and without T2D. (**A**) Exploratory Pearson correlations between metabolic syndrome parameters and metabolic variables in MS and NMR selected from the mouse study. *p*-values were not corrected for multiple comparisons. (**B**–**E**) Examples of correlation plots between metabolic syndrome parameters and NMR/MS variables. Abbreviations: BMI, body mass index; CRP, C-reactive protein; DMGV, Dimethylguanidino valeric acid; DBP, diastolic blood pressure; HDL, high-density lipoprotein; 2-HIV, 2-Hydroxyisovalerate; HOMA-IR, homeostatic model assessment for insulin resistance; LDL, low-density lipoprotein; SAH, S-Adenosyl-L-homocysteine; SBP, systolic blood pressure; TAG, triacylglycerols.

Finally, we set to conduct a cross-sectional analysis of MDCS data for 34 NMR chemical shifts and MS-measured metabolites. These variables were selected based on the magnitude of concentration changes after either 1 or 8 weeks of HFD exposure in mice, as ascertained from significance in t-tests applied (Supplementary Figure S5). From those metabolites, not all were detectable in human plasma. Correlation analysis revealed associations between metabolite levels and metabolic syndrome parameters (Figure 6). Interestingly, impaired glucose homeostasis, which was depicted by glycosylated haemoglobin (HbA1c), glucose, insulin and/or HOMA-IR, correlated positively with plasma glucose concentrations and negatively with taurine, S-adenosylhomocysteine (SAH), NADP^+^, caprylate, arginine, alanine and 2-HIV. Although less significant, obesity depicted by body mass index (BMI) and/or waist circumference correlated positively with levels of lactate, kynurenine, homoarginine, glucose and dimethylguanidino valeric acid (DMGV), and negatively with 2-hydroxyisovalerate (2-HIV). Dyslipidaemia (levels of cholesterol, TAG, HDL and LDL) also showed correlations with the concentrations of trimethyllysine, taurine, SAH, L-targinine, kynurenine, hippurate, glucose, DMGV and 2-HIV.

## 4. Discussion

The combination of MS and NMR spectroscopy has been proposed to improve the overall quality of metabolomic studies by augmenting the coverage of the metabolome, although hitherto there are limited studies effectively combining the two analytical methods for metabolomics in the medical field [28,29,30]. In the present work, we demonstrate that the NMR-MS combined approach improves the sensitivity for determining plasma metabolome alterations in a diet-induced obesity mouse model that develops metabolic syndrome. In fact, OPLS regression applied to NMR and MS data together resulted in improved group separation regarding both HFD exposure, and time-on-diet (or age), relative to analysis of data from each analytical technique separately (see Figure 2). The top three metabolites that appeared to drive metabolic syndrome during HFD exposure were xanthine, hippurate and 3-hydroxyisobutyrate.

After 1 week of HFD exposure, xanthine concentration already appeared decreased in the mouse plasma. In contrast to our finding, HFD exposure in mice was reported to increase xanthine levels in urine of obesity-prone but not obesity-resistant mice (Wei et al., 2021). Xanthine is a diet-derived compound, namely following the metabolism of compounds like caffeine, theobromine and theophylline, and is also considered to be a host-microbiome co-metabolite. Accordingly, Wei et al. proposed that elevated urinary xanthine was triggered by increased abundance of gut bacteria of the genera *Bifidobacteriaceae*, *Roseburia* and *Escherichia* [31]. However, none of these genera was found to associate with xanthine in human plasma in the GutsyAtlas data [32]. The reduced xanthine concentrations upon HFD exposure are more in line with reports of increased activity of the purine catabolism enzymes xanthine oxidoreductase and xanthine oxidase in serum of individuals with obesity, metabolic syndrome and T2D [33,34,35]. In our study, pathway analysis indicated altered purine metabolism at both 1 and 8 weeks of HFD exposure in mice, and also in individuals with T2D. While xanthine was not observed in plasma samples obtained from the MDCS cohort, targeted analysis of hypoxanthine and paraxanthine were found to be correlated with metabolic syndrome parameters (see Figure 6).

Plasma hippurate concentrations were found to be reduced by HFD exposure, independently of the exposure duration. In contrast, they did not seem to be associated with T2D prevalence in the MDCS cohort (VIP = 0.449), and, in a targeted analysis, hippurate was positively correlated with plasma levels of cholesterol and LDL (Figure 6). These contrasting results might relate to microbiome differences in the human cohort and the laboratory mouse. Indeed, hippurate was suggested as a metabolomic marker of gut microbiome diversity and associated with increased risk of metabolic syndrome [36,37]. Interestingly, treatment of HFD-fed mice with hippurate at 20 nmol/day was reported to improve glucose tolerance and to augment glucose-induced insulin secretion [37].

Levels of the valine derivate 2-hydroxyisovalerate (2-HIV) increased during HFD exposure, and metabolism of branched-chain amino acids was significantly impacted by HFD in pathway analysis. The concentration of another intermediary metabolite of valine degradation, 3-hydroxyisobutyrate (3-HIB), was reduced after 1 week of HFD, but increased after 8 weeks of HFD feeding (VIP = 1.286). These metabolite alterations are in line with the known involvement of branched-chain amino acids in metabolic syndrome and diabetes [38], which our pathway analysis found altered in both HFD-fed mice and diabetes individuals. In a targeted analysis of variables from the MDCS cohort, 2-HIV was found to be inversely correlated with metabolic syndrome at level of both glucose and lipid homeostasis. In line with our findings, plasma 2-HIV levels were found to be associated with BMI, while urinary levels of 2-HIV were associated with glycemic control [39,40]. Cobb et al. [41] and Yousri et al. [40] reported an association between 3-HIB and glycemic control, and Mardinoglu et al. [42] reported an association of plasma 3-HIB levels with T2D. Moreover, this metabolite appears to be an adipocyte metabolism regulator and to be involved in obesity development, since it modulates insulin-dependent glucose uptake, increases fatty acid accumulation and reduces mitochondrial respiration [43].

*S*-Adenosylhomocysteine (SAH) was found to be increased in HFD-fed mice at both 1 and 8 weeks. Furthermore, in the targeted analysis of the MDCS cohort, SAH seemed associated with a healthy metabolic phenotype, since it negatively correlated with parameters of impaired glucose homeostasis and dyslipidemia (see Figure 6). SAH is produced during methylation reactions, and it can be hydrolysed to adenosine and homocysteine [44]. Adenosine is acknowledged to have cytoprotective properties, namely by acting on the abundant adenosine A_1_ receptors; alterations of adenosinergic signalling have been implicated in diabetes, and modulation of the adenosinergic system has been proposed as health promotion strategy [45]. In turn, homocysteine is found increased in metabolic disease and type 2 diabetes [46] and particularly associated with cardiovascular disease [47]. However, healthy effects of higher SAH might be rather related to mimicking physiological effects of caloric restriction by stimulating *S*-adenosylmethionine synthesis, which was proposed for *Saccharomyces cerevisiae* [48].

We conducted similar OPLS analyses in samples from the animal model and from the human cohort. In the latter, not surprisingly, the top VIP scores were dominated by NMR chemical shifts of glucose. Nevertheless, we identified more than 200 NMR and MS variables with VIP > 1 for identifying T2D, which then revealed alterations in about 40 metabolic pathways of the KEGG database. Metabolic pathways deemed significantly impacted by HFD feeding in mice were also significantly altered in individuals with diabetes, when compared to healthy controls. Other metabolites were found important in the separation of mouse groups upon experimentally induced obesity, and their plasma concentrations eventually showed correlation with metabolic syndrome parameters in the MDCS cohort. For example, our targeted exploratory analysis of the MDCS cohort showed a correlation between levels of dimethylguanidino valeric acid (DMGV), obesity and dyslipidemia, which confirms the previously reported association with hepatic fat accumulation [49]. Interestingly, DMGV is also associated with some degree of resistance to metabolic benefits of physical exercise [50]. In our point of view, a strength of this study is the translational approach that allows us to test the findings from the mouse study in plasma obtained from the MDCS cohort.

An important limitation of our study is the sample size. However, this study was meant be an exploration of the potential of the NMR-MS combined approach for metabolomics in metabolic syndrome and diabetes, which was successfully demonstrated. Furthermore, the mouse study has focused only on male mice, which precludes the analysis of sex-specific differences in metabolic regulation and disease susceptibility. However, the inclusion of both sexes would reduce the statistical power of this exploratory study due to the important sex dimorphism that we have observed in diet-induced obesity in mice [13,14,15].

Typically, binning of spectral segments is employed in the spectra processing pipeline, thus reducing the number of variables for subsequent analysis [51]. This approach might result in the loss of spectral resolution, and most importantly, loss of small signals in the spectra. Therefore, we have opted for unbiased peak detection (local maxima) in the sum of all spectra after chemical shift alignment. Using this approach, it is likely that peaks from metabolites existing in small amounts are preserved for further analysis.

A disadvantage of ^1^H NMR spectroscopy is the complex metabolite signals and their substantial overlap in recorded spectra. Therefore, rather than signal quantification, we opted for an unbiased analysis of the spectral signature, thus enhancing the number of variables for pattern recognition in OPLS. However, overlapping peaks were not used for pathway analysis since one cannot determine which metabolite resulted in the observed signal change. In future studies, this could be improved by acquiring 2D NMR spectra using experiments such as total correlation spectroscopy (^1^H-^1^H TOCSY), which would provide additional spin-system information for assigning peaks to metabolites.

## 5. Conclusions

Altogether, we conclude that a combined, unbiased NMR+MS approach is relevant for metabolomics studies in the realm of metabolic disease. Despite being limited by the small sample size of this study, this approach allowed us to better define metabolic imbalances caused by HFD exposure in mice as early after 1 week of dietary intervention (likely before end-organ damage is established). All metabolic alterations observed in HFD-fed mice and many more were confirmed in individuals with diabetes.

## Figures and Tables

**Table 1:** Characteristics of subjects in the present study.

	No Diabetes (n = 23)	Diabetes (n = 23)	*p*-Value
Sex, female n (%)	15 (65%)	14 (61%)	n.s.
Obesity, BMI > 25 n (%)	2 (9%)	9 (39%)	0.016
Age (years)	60 ± 6	61 ± 5	n.s.
BMI (kg/m^2^)	25.2 ± 3.4	29.4 ± 5.9	0.015
Waist circumference (cm)	80 ± 11	95 ± 14	<0.001
Systolic BP (mm Hg)	152 ± 18	156 ± 23	n.s.
Diastolic BP (mm Hg)	90 ± 8	90 ± 9	n.s.
Fasting glucose (mmol/L)	5.2 ± 0.5	9.3 ± 3.3	<0.001
Fasting Insulin (µU/mL)	6.4 ± 3.1	13.2 ± 7.6	<0.001
HOMA-IR	1.6 ± 1.0	5.0 ± 3.3	<0.001
HbA1c (%)	4.9 ± 0.4	7.2 ± 1.9	<0.001
Cholesterol (mmol/L)	6.3 ± 1.1	6.1 ± 0.6	n.s.
Triglycerides (mmol/L)	1.4 ± 0.8	1.9 ± 1.0	n.s.
HDL (mmol/L)	1.3 ± 0.3	1.2 ± 0.3	n.s.
LDL (mmol/L)	4.3 ± 1.0	4.1 ± 0.7	n.s.
CRP (mgl/L)	0.31 ± 0.36	0.48 ± 0.32 ^a^	0.025

Data is mean ± SD, unless otherwise stated; ^a^ 2 samples missing; abbreviations: BMI, body-mass index; BP, blood pressure; CRP, C-reactive protein; HbA1c, glycated hemoglobin; HDL, high-density lipoprotein; HOMA-IR, Homeostatic Model Assessment for Insulin Resistance; LDL, low-density lipoprotein. *p*-values were estimated from Mann–Whitney or χ^2^ tests, where appropriate. *p* > 0.05 denoted as non-significant (n.s.).

## Data Availability

The data presented in this study are available on request from the corresponding author. The data are not publicly available due to ethical and privacy reasons.

## Supplementary Materials

The following supporting information can be downloaded at: https://www.mdpi.com/article/10.3390/metabo13070874/s1, Document S1: output from MetaboAnalyst analysis of mouse experiments; Document S2. output from MetaboAnalyst analysis of individuals with and without diabetes; Table S1. List of reported metabolites from MS measurements; Figure S1. NMR spectroscopy of human plasma at 600 MHz. The solid line represents the measured spectrum, and the circles depict the data points taken for OPLS analysis; Figure S2. NMR spectroscopy of mouse plasma at 500 MHz. The solid line represents the measured spectrum, and the circles depict the data points taken for OPLS analysis; Figure S3. MetaboAnalyst results using SMPDB for HFD vs. CD in mouse experiments. Analysis was performed on z-scores from metabolites with VIP > 1 in OPLS regression for combined MS and NMR spectroscopy data. Top graphs show the pathway impact determined using MetaboAnalyst and respective significance (*p*-values). Pathways with impact score = 0 are not represented. Dashed lines indicate *p* = 0.05. Bottom graphs include significant findings, that is, pathways with unadjusted *p* < 0.05. Orange symbols and blue bars represent number of hits (metabolites analysed in that pathway) and –log10(P), respectively. Bars highlighted with black border indicate pathways which significance survives false discovery rate (FDR) correction; Figure S4. Comparison of MetaboAnalyst analysis of HFD vs. CD with the KEGG library using MS+NMR data, MS data alone and NMR data alone. Orange symbols and blue bars represent number of hits (metabolites analysed in that pathway) and −log10(P), respectively. Bars highlighted with black border indicate pathways in which significance survives false discovery rate (FDR) correction; Figure S5. MetaboAnalyst results using SMPDB for diabetes vs. no diabetes in MDCS samples. Analysis was performed on z-scores from metabolites with VIP > 1 in OPLS regression for combined MS and NMR spectroscopy data. The graphs on the left show the pathway impact determined using MetaboAnalyst and respective significance (*p*-values). Pathways with impact score = 0 are not represented; the dashed line indicates *p* = 0.05. Graph on the right includes pathways with adjusted *p* < 0.05 (after false discovery rate correction). Orange symbols and blue bars represent number of hits (metabolites analysed in that pathway) and −log10(P), respectively; Figure S6. Volcano plots resulting from t-tests comparing z-scores for HFD vs. CD.

## References

[1] Blüher M. (2019). Obesity: Global epidemiology and pathogenesis. Nat. Rev. Endocrinol..

[2] Casadei-Gardini A., Coco L.D., Marisi G., Conti F., Rovesti G., Ulivi P., Canale M., Frassineti G.L., Foschi F.G., Longo S. (2020). ^1^H-NMR based serum metabolomics highlights different specific biomarkers between early and advanced hepatocellular carcinoma stages. Cancers.

[3] Clish C.B. (2015). Metabolomics: An emerging but powerful tool for precision medicine. Mol. Case Stud..

[4] Cirulli E.T., Guo L., Leon Swisher C., Shah N., Huang L., Napier L.A., Kirkness E.F., Spector T.D., Caskey C.T., Thorens B. (2019). Profound Perturbation of the Metabolome in Obesity Is Associated with Health Risk. Cell Metab..

[5] Smith E., Fernandez C., Melander O., Ottosson F. (2020). Altered acylcarnitine metabolism is associated with an increased risk of atrial fibrillation. J. Am. Heart Assoc..

[6] Smith E., Ericson U., Hellstrand S., Orho-Melander M., Nilsson P.M., Fernandez C., Melander O., Ottosson F. (2022). A healthy dietary metabolic signature is associated with a lower risk for type 2 diabetes and coronary artery disease. BMC Med..

[7] Emwas A.H., Roy R., McKay R.T., Tenori L., Saccenti E., Nagana Gowda G.A., Raftery D., Alahmari F., Jaremko L., Jaremko M. (2019). Nmr spectroscopy for metabolomics research. Metabolites.

[8] Nagana Gowda G.A., Gowda Y.N., Raftery D. (2015). Expanding the limits of human blood metabolite quantitation using NMR spectroscopy. Anal. Chem..

[9] Deidda M., Piras C., Bassareo P.P., Cadeddu Dessalvi C., Mercuro G. (2015). Metabolomics, a promising approach to translational research in cardiology. IJC Metab. Endocr..

[10] Nagana Gowda G.A., Raftery D. (2017). Whole Blood Metabolomics by 1H NMR Spectroscopy Provides a New Opportunity to Evaluate Coenzymes and Antioxidants. Anal. Chem..

[11] Soares A.F., Duarte J.M.N., Gruetter R. (2018). Increased hepatic fatty acid polyunsaturation precedes ectopic lipid deposition in the liver in adaptation to high-fat diets in mice. Magma.

[12] Berglund G., Elmstähl S., Janzon L., Larsson S.A. (1993). The Malmo Diet and Cancer Study. Design and feasibility. J. Intern. Med..

[13] Lizarbe B., Soares A.F., Larsson S., Duarte J.M.N. (2019). Neurochemical Modifications in the Hippocampus, Cortex and Hypothalamus of Mice Exposed to Long-Term High-Fat Diet. Front. Neurosci..

[14] Garcia-Serrano A.M., Mohr A.A., Philippe J., Skoug C., Spégel P., Duarte J.M.N. (2022). Cognitive Impairment and Metabolite Profile Alterations in the Hippocampus and Cortex of Male and Female Mice Exposed to a Fat and Sugar-Rich Diet are Normalized by Diet Reversal. Aging Dis..

[15] Garcia-Serrano A.M., Vieira J.P.P., Fleischhart V., Duarte J.M.N. (2022). Taurine and *N*-acetylcysteine treatments prevent memory impairment and metabolite profile alterations in the hippocampus of high-fat diet-fed female mice. Nutr. Neurosci..

[16] Castañar L., Poggetto G.D., Colbourne A.A., Morris G.A., Nilsson M. (2018). The GNAT: A new tool for processing NMR data. Magn. Reson. Chem..

[17] Ala-Korpela M. (1995). ^1^H NMR spectroscopy of human blood plasma. Prog. Nucl. Magn. Reson. Spectrosc..

[18] McHugh C.E., Flott T.L., Schooff C.R., Smiley Z., Puskarich M.A., Myers D.D., Younger J.G., Jones A.E., Stringer K.A. (2018). Rapid, reproducible, quantifiable nmr metabolomics: Methanol and methanol: Chloroform precipitation for removal of macromolecules in serum and whole blood. Metabolites.

[19] Nagana Gowda G.A., Raftery D. (2014). Quantitating metabolites in protein precipitated serum using NMR spectroscopy. Anal. Chem..

[20] Nagana Gowda G.A., Raftery D. (2021). NMR-Based Metabolomics. Adv. Exp. Med. Biol..

[21] Wishart D.S., Feunang Y.D., Marcu A., Guo A.C., Liang K., Vázquez-Fresno R., Sajed T., Johnson D., Li C., Karu N. (2018). HMDB 4.0: The human metabolome database for 2018. Nucleic Acids Res..

[22] Guijas C., Montenegro-Burke J.R., Domingo-Almenara X., Palermo A., Warth B., Hermann G., Koellensperger G., Huan T., Uritboonthai W., Aisporna A.E. (2018). METLIN: A Technology Platform for Identifying Knowns and Unknowns. Anal. Chem..

[23] Sumner L.W., Amberg A., Barrett D., Beale M.H., Beger R., Daykin C.A., Fan T.W.-M., Fiehn O., Goodacre R., Griffin J.L. (2007). Proposed minimum reporting standards for chemical analysis Chemical Analysis Working Group (CAWG) Metabolomics Standards Initiative (MSI). Metabolomics.

[24] Dunn W.B., Broadhurst D., Begley P., Zelena E., Francis-McIntyre S., Anderson N., Brown M., Knowles J.D., Halsall A., Haselden J.N. (2011). Procedures for large-scale metabolic profiling of serum and plasma using gas chromatography and liquid chromatography coupled to mass spectrometry. Nat. Protoc..

[25] Pang Z., Chong J., Zhou G., De Lima Morais D.A., Chang L., Barrette M., Gauthier C., Jacques P.É., Li S., Xia J. (2021). MetaboAnalyst 5.0: Narrowing the gap between raw spectra and functional insights. Nucleic Acids Res..

[26] Frolkis A., Knox C., Lim E., Jewison T., Law V., Hau D.D., Liu P., Gautam B., Ly S., Guo A.C. (2010). SMPDB: The Small Molecule Pathway Database. Nucleic Acids Res..

[27] Jewison T., Su Y., Disfany F.M., Liang Y., Knox C., Maciejewski A., Poelzer J., Huynh J., Zhou Y., Arndt D. (2014). SMPDB 2.0: Big improvements to the Small Molecule Pathway Database. Nucleic Acids Res..

[28] Marshall D.D., Powers R. (2017). Beyond the paradigm: Combining mass spectrometry and nuclear magnetic resonance for metabolomics. Prog Nucl. Magn. Reason. Spectrosc..

[29] Letertre M.P.M., Giraudeau P., de Tullio P. (2021). Nuclear Magnetic Resonance Spectroscopy in Clinical Metabolomics and Personalized Medicine: Current Challenges and Perspectives. Front. Mol. Biosci..

[30] Letertre M.P.M., Dervilly G., Giraudeau P. (2021). Combined Nuclear Magnetic Resonance Spectroscopy and Mass Spectrometry Approaches for Metabolomics. Anal. Chem..

[31] Wei B., Wang S., Wang Y., Ke S., Jin W., Chen J., Zhang H., Sun J., Henning S.M., Wang J. (2021). Gut microbiota-mediated xanthine metabolism is associated with resistance to high-fat diet-induced obesity. J. Nutr. Biochem..

[32] Dekkers K.F., Sayols-Baixeras S., Baldanzi G., Nowak C., Hammar U., Nguyen D., Varotsis G., Brunkwall L., Nielsen N., Eklund A.C. (2022). An online atlas of human plasma metabolite signatures of gut microbiome composition. Nat. Commun..

[33] Miric D.J., Kisic B.M., Filipovic-Danic S., Grbic R., Dragojevic I., Miric M.B., Puhalo-Sladoje D. (2016). Xanthine Oxidase Activity in Type 2 Diabetes Mellitus Patients with and without Diabetic Peripheral Neuropathy. J. Diabetes Res..

[34] Okuyama T., Shirakawa J., Nakamura T., Murase T., Miyashita D., Inoue R., Kyohara M., Togashi Y., Terauchi Y. (2021). Association of the plasma xanthine oxidoreductase activity with the metabolic parameters and vascular complications in patients with type 2 diabetes. Sci. Rep..

[35] Hernandez-Hernandez M.E., Torres-Rasgado E., Pulido-Perez P., Nicolás-Toledo L., Martínez-Gómez M., Rodríguez-Antolín J., Pérez-Fuentes R., Romero J.R. (2022). Disordered Glucose Levels Are Associated with Xanthine Oxidase Activity in Overweight Type 2 Diabetic Women. Int. J. Mol. Sci..

[36] Pallister T., Jackson M.A., Martin T.C., Zierer J., Jennings A., Mohney R.P., MacGregor A., Steves C.J., Cassidy A., Spector T.D. (2017). Hippurate as a metabolomic marker of gut microbiome diversity: Modulation by diet and relationship to metabolic syndrome. Sci. Rep..

[37] Brial F., Chilloux J., Nielsen T., Vieira-Silva S., Falony G., Andrikopoulos P., Olanipekun M., Hoyles L., Djouadi F., Neves A.L. (2021). Human and preclinical studies of the host-gut microbiome co-metabolite hippurate as a marker and mediator of metabolic health. Gut.

[38] Vanweert F., Schrauwen P., Phielix E. (2022). Role of branched-chain amino acid metabolism in the pathogenesis of obesity and type 2 diabetes-related metabolic disturbances BCAA metabolism in type 2 diabetes. Nutr. Diabetes.

[39] Yousri N.A., Suhre K., Yassin E., Al-Shakaki A., Robay A., Elshafei M., Chidiac O., Hunt S.C., Crystal R.G., Fakhro K.A. (2022). Metabolic and Metabo-Clinical Signatures of Type 2 Diabetes, Obesity, Retinopathy, and Dyslipidemia. Diabetes.

[40] Yousri N.A., Mook-Kanamori D.O., Selim M.M., Takiddin A.H., Al-Homsi H., Al-Mahmoud K.A., Karoly E.D., Krumsiek J., Do K.T., Neumaier U. (2015). A systems view of type 2 diabetes-associated metabolic perturbations in saliva, blood and urine at different timescales of glycaemic control. Diabetologia.

[41] Cobb J., Eckhart A., Motsinger-Reif A., Carr B., Groop L., Ferrannini E. (2016). α-Hydroxybutyric Acid Is a Selective Metabolite Biomarker of Impaired Glucose Tolerance. Diabetes Care.

[42] Mardinoglu A., Gogg S., Lotta L.A., Stančáková A., Nerstedt A., Boren J., Blüher M., Ferrannini E., Langenberg C., Wareham N.J. (2018). Elevated Plasma Levels of 3-Hydroxyisobutyric Acid Are Associated With Incident Type 2 Diabetes. EBioMedicine.

[43] Nilsen M.S., Jersin R.Å., Ulvik A., Madsen A., McCann A., Svensson P.A., Svensson M.K., Nedrebø B.G., Gudbrandsen O.A., Tell G.S. (2020). 3-Hydroxyisobutyrate, A Strong Marker of Insulin Resistance in Type 2 Diabetes and Obesity That Modulates White and Brown Adipocyte Metabolism. Diabetes.

[44] Vizán P., Di Croce L., Aranda S. (2021). Functional and Pathological Roles of AHCY. Front. Cell Dev. Biol..

[45] Antonioli L., Blandizzi C., Csóka B., Pacher P., Haskó G. (2015). Adenosine signalling in diabetes mellitus--pathophysiology and therapeutic considerations. Nature reviews. Endocrinology.

[46] Huang T., Ren J., Huang J., Li D. (2013). Association of homocysteine with type 2 diabetes: A meta-analysis implementing Mendelian randomization approach. BMC Genom..

[47] Herrmann W., Herrmann M. (2022). The Controversial Role of HCY and Vitamin B Deficiency in Cardiovascular Diseases. Nutrients.

[48] Ogawa T., Tsubakiyama R., Kanai M., Koyama T., Fujii T., Iefuji H., Soga T., Kume K., Miyakawa T., Hirata D. (2016). Stimulating S-adenosyl-l-methionine synthesis extends lifespan via activation of AMPK. Proc. Natl. Acad. Sci. USA.

[49] O’Sullivan J.F., Morningstar J.E., Yang Q., Zheng B., Gao Y., Jeanfavre S., Scott J., Fernandez C., Zheng H., O’Connor S. (2017). Dimethylguanidino valeric acid is a marker of liver fat and predicts diabetes. J. Clin. Investig..

[50] Robbins J.M., Herzig M., Morningstar J., Sarzynski M.A., Cruz D.E., Wang T.J., Gao Y., Wilson J.G., Bouchard C., Rankinen T. (2019). Association of Dimethylguanidino Valeric Acid With Partial Resistance to Metabolic Health Benefits of Regular Exercise. JAMA Cardiol..

[51] Emwas A.H., Saccenti E., Gao X., McKay R.T., dos Santos V.A.P.M., Roy R., Wishart D.S. (2018). Recommended strategies for spectral processing and post-processing of 1D 1 H-NMR data of biofluids with a particular focus on urine. Metabolomics.

