# Supplementary material for: Metabolite Profiling in a Diet-Induced Obesity Mouse Model and Individuals with Diabetes: A Combined Mass Spectrometry and Proton Nuclear Magnetic Resonance Spectroscopy Study
Source: Metabolites. 2023 Jul 23;13(7):874. doi: 10.3390/metabo13070874 (PMC10385288; doi:10.3390/metabo13070874)
Supplement: Supplementary file 1 [file metabolites-13-00874-s001.zip › Supplementary Table S1.pdf]

Table S1. List of reported metabolites from MS measurements.

In total, 108 Identified and putatively annotated metabolites were measured. A list of reported metabolites can be found below, including corresponding m/z, chromatographic retention time, HMDB identifier and annotation confidence. Annotations with confidence level 1 were confirmed using an in-house metabolite library of synthetic standards.

**Metabolites**	**m/z**	**rt**	**HMDB ID**	**Annotation Level**
Alanine	90.0553	7.5	HMDB00161	1
Dimethylglycine	104.0703	6.59	HMDB00092	1
Choline	104.1074	4.01	HMDB00097	1
Creatinine	114.0663	4.55	HMDB00562	1
Proline	116.0703	6.64	HMDB00162	1
Guanidineacetate	118.0613	7.56	HMDB00128	1
Betaine	118.0864	6.14	HMDB00043	1
Threonine	120.0653	7.85	HMDB00167	1
Nicotinamide	123.0553	1.36	HMDB01406	1
Taurine	126.0223	6.62	HMDB00251	1
Pyroglutamate	130.0503	2.56	HMDB00267	2
N-methylproline	130.0863	6.25	HMDB94696	1
Pipecolate	130.0863	6.84	HMDB00716	1
Creatine	132.0773	7.19	HMDB00064	1
Isoleucine	132.1023	6.09	HMDB00172	1
Leucine	132.1023	5.92	HMDB00687	1
Asparagine	133.0603	8.7	HMDB00168	1
Ornithine	133.0973	11.58	HMDB00214	1
Trigonelline	137.0474	6.38	HMDB00875	1
Methylnicotinamide	137.0704	4.96	HMDB03152	1
Proline.betaine	144.1023	6.21	HMDB04827	1
4-trimethylammoniobutanoate	146.1174	4.58	HMDB01161	1
Glutamine	147.0763	8.48	HMDB00641	1
Lysine	147.1133	11.4	HMDB00182	1
Glutamate	148.0603	8.14	HMDB00148	1
Methionine	150.0583	6.32	HMDB00696	1
N−Methyl−4−pyridone−3−carboxamide	153.0653	1.92	HMDB04194	1
Methyllysine	161.1283	10.66	HMDB02038	1
Carnitine	162.1124	6.19	HMDB00062	1
Methionine-S-oxide	166.0533	8.23	HMDB02005	1
7-methylguanine	166.0723	4.77	HMDB00897	1
Phenylalanine	166.0863	5.88	HMDB00159	1
3-methylhistidine	170.0923	10.82	HMDB00479	1
Acetylornithine	175.1093	7.86	HMDB03357	1
Arginine	175.1193	10.92	HMDB00517	1
Citrulline	176.1023	9.04	HMDB00904	1
Dimethyllysine	176.1513	10.17	0	1

Cotinine	177.1023	1.43	HMDB0001046	1
Tyrosine	182.0813	6.64	HMDB00158	1
Homoarginine	189.1343	10.59	HMDB00670	1
Kynurenate	190.0503	5.06	HMDB00715	1
Homocitrulline	190.1183	8.86	HMDB00679	1
DMGV	202.1184	6.01	HMDB0240212	1
ADMA	203.1503	9.52	HMDB01539	1
Acylcarnitine C2:0	204.1234	4.45	HMDB00201	1
Tryptophan	205.0973	5.93	HMDB00929	1
3-hydroxytrimethyllysine	205.1544	10.8	HMDB01422	1
Kynurenine	209.0923	6	HMDB00684	1
Acetylarginine	217.1293	7.08	HMDB04620	1
Acylcarnitine C3:0	218.1384	4.06	HMDB00824	1
Ergothioneine	230.0953	7.35	HMDB03045	1
Acylcarnitine C4:0	232.1534	3.63	HMDB02013	1
Tiglylcarnitine	243.1464	3.46	HMDB02366	2
Acylcarnitine C5:0	245.1624	3.23	HMDB00688	1
Glycerophosphocholine	259.1173	8.71	HMDB00086	2
Acylcarnitine C6:0	260.1854	2.88	HMDB00756	2
Phenylacetylglutamine	265.1173	1.98	HMDB06344	1
1-methyladenosine	282.1203	6.46	HMDB03331	2
Acylcarnitine C8:0	288.2164	2.49	HMDB00791	1
Acylcarnitine C10:3	310.2014	2.42	0	2
Acylcarnitine C10:2	312.2164	2.44	0	2
Acylcarnitine C10:1	314.2314	2.34	HMDB13205	2
Acylcarnitine C10:0	316.2484	2.18	HMDB00651	1
Acylcarnitine C12:1	342.2624	2.16	HMDB13326	2
Acylcarnitine C12:0	344.2784	2.13	HMDB02250	2
Acylcarnitine C14:1	370.2934	2.03	HMDB0240588	2
Acylcarnitine C14:0	372.3104	1.94	HMDB05066	1
Acylcarnitine C16:0	400.3424	1.85	HMDB00222	1
25-hydroxyvitamin-D3	401.3413	0.81	HMDB03550	1
Acylcarnitine C18:2	424.3454	1.87	0	2
Acylcarnitine C18:1	426.3564	1.86	0	2
Beta-carotene	537.4453	0.83	HMDB00561	1
