# Supplementary figures and images for: Metabolite Profiling in a Diet-Induced Obesity Mouse Model and Individuals with Diabetes: A Combined Mass Spectrometry and Proton Nuclear Magnetic Resonance Spectroscopy Study
Source: Metabolites. 2023 Jul 23;13(7):874. doi: 10.3390/metabo13070874 (PMC10385288; doi:10.3390/metabo13070874)

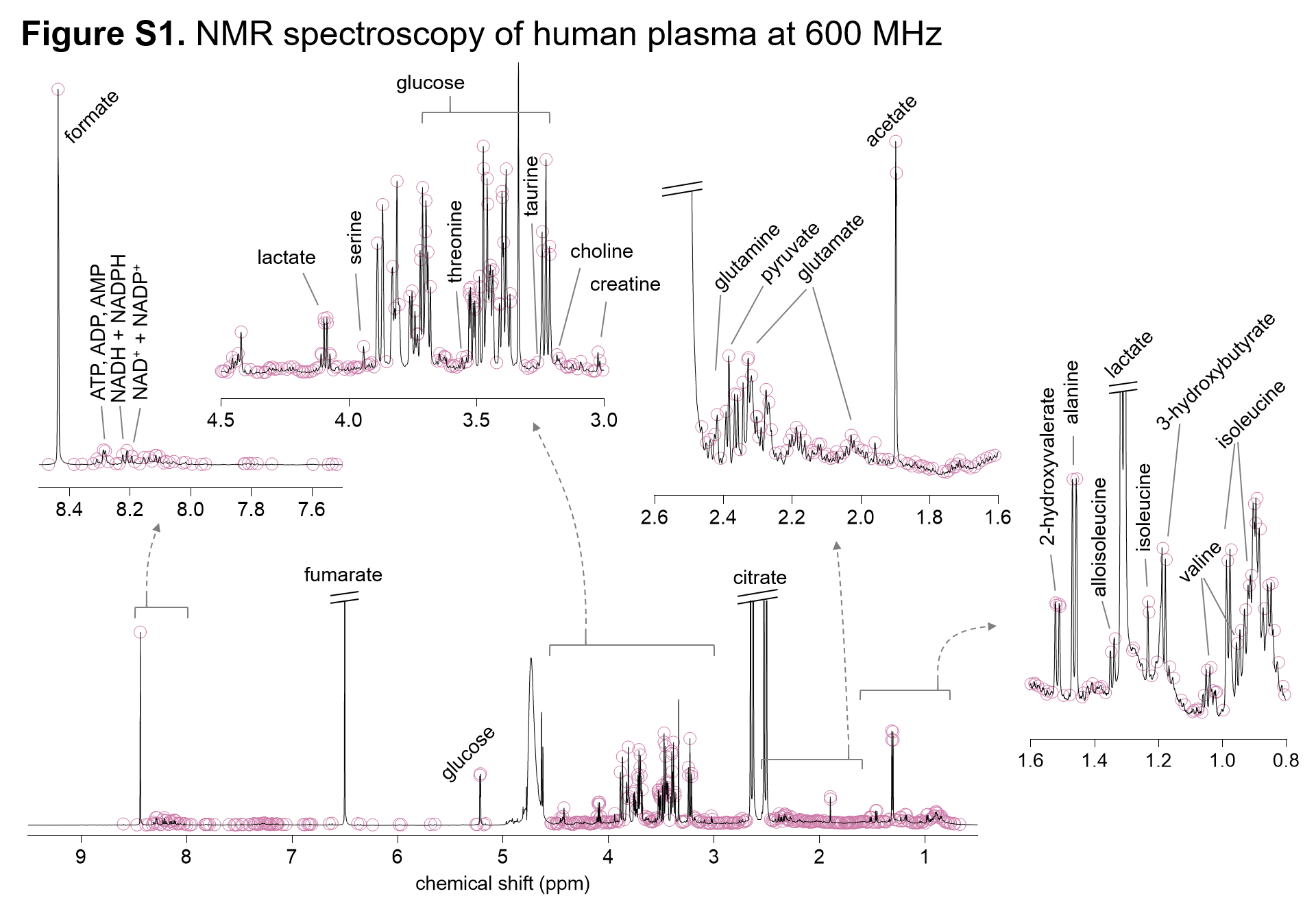

Supplement: Supplementary file 1 [file metabolites-13-00874-s001.zip › Figure S1.tif]

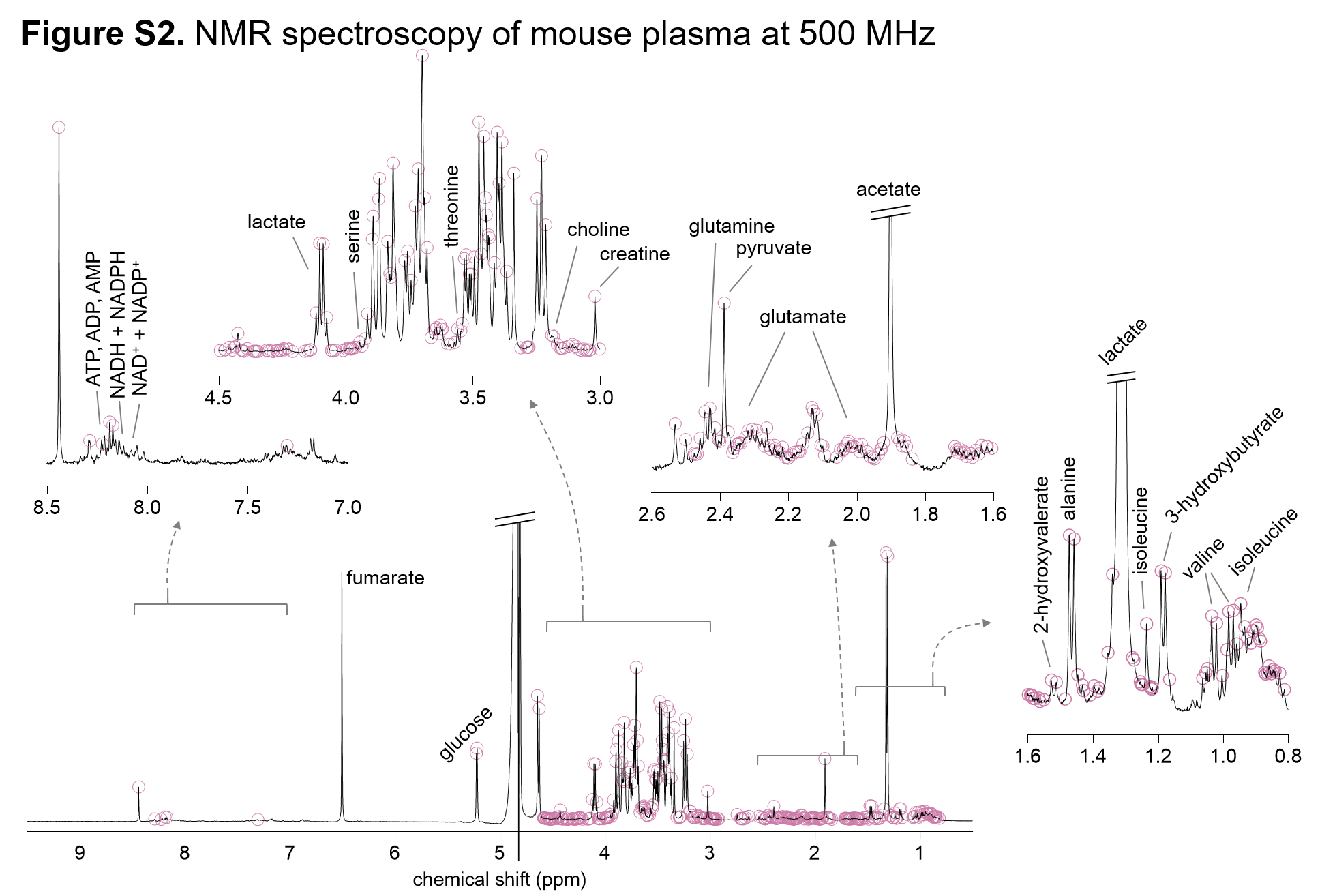

Supplement: Supplementary file 1 [file metabolites-13-00874-s001.zip › Figure S2.tif]

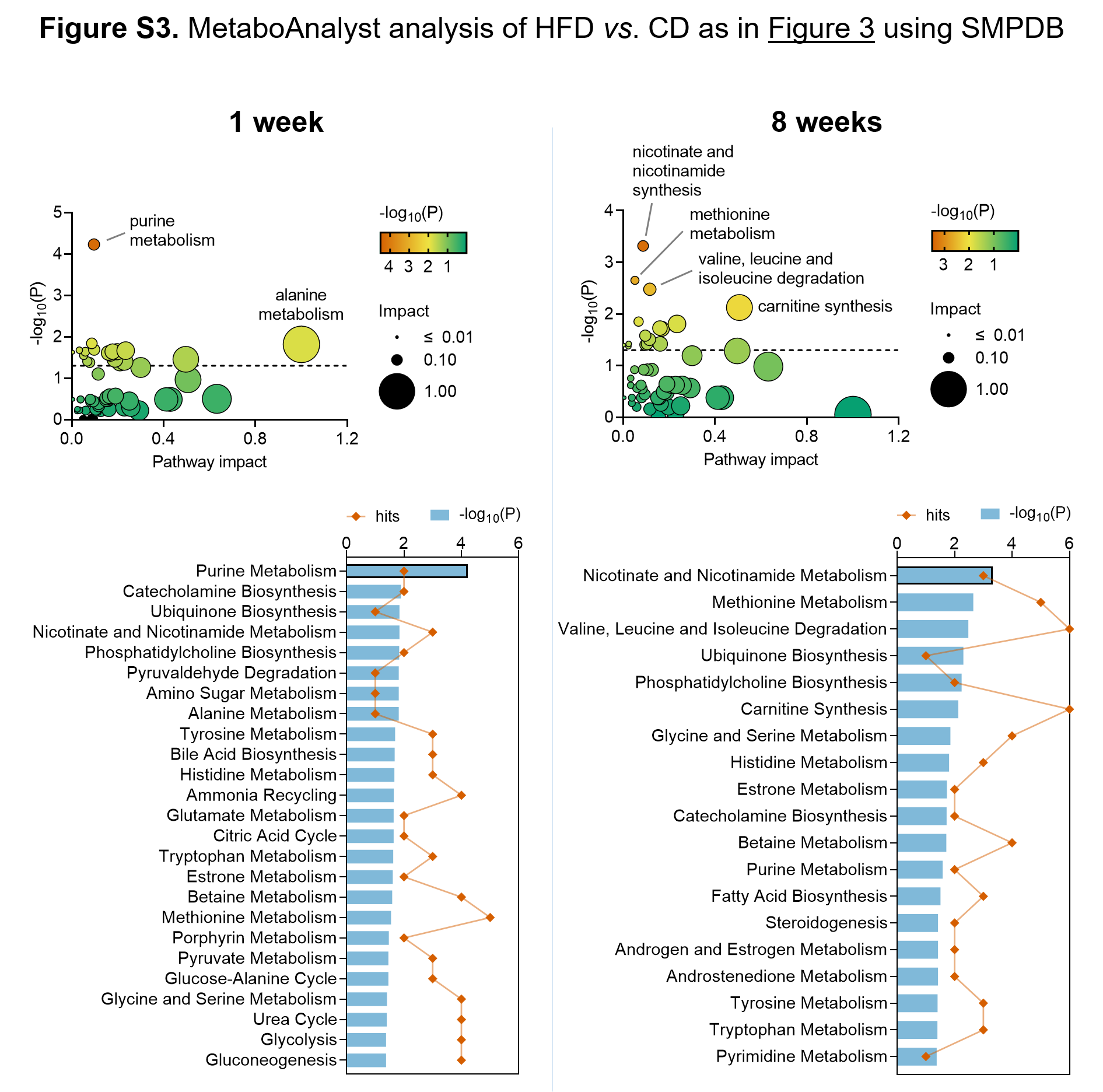

Supplement: Supplementary file 1 [file metabolites-13-00874-s001.zip › Figure S3.tif]

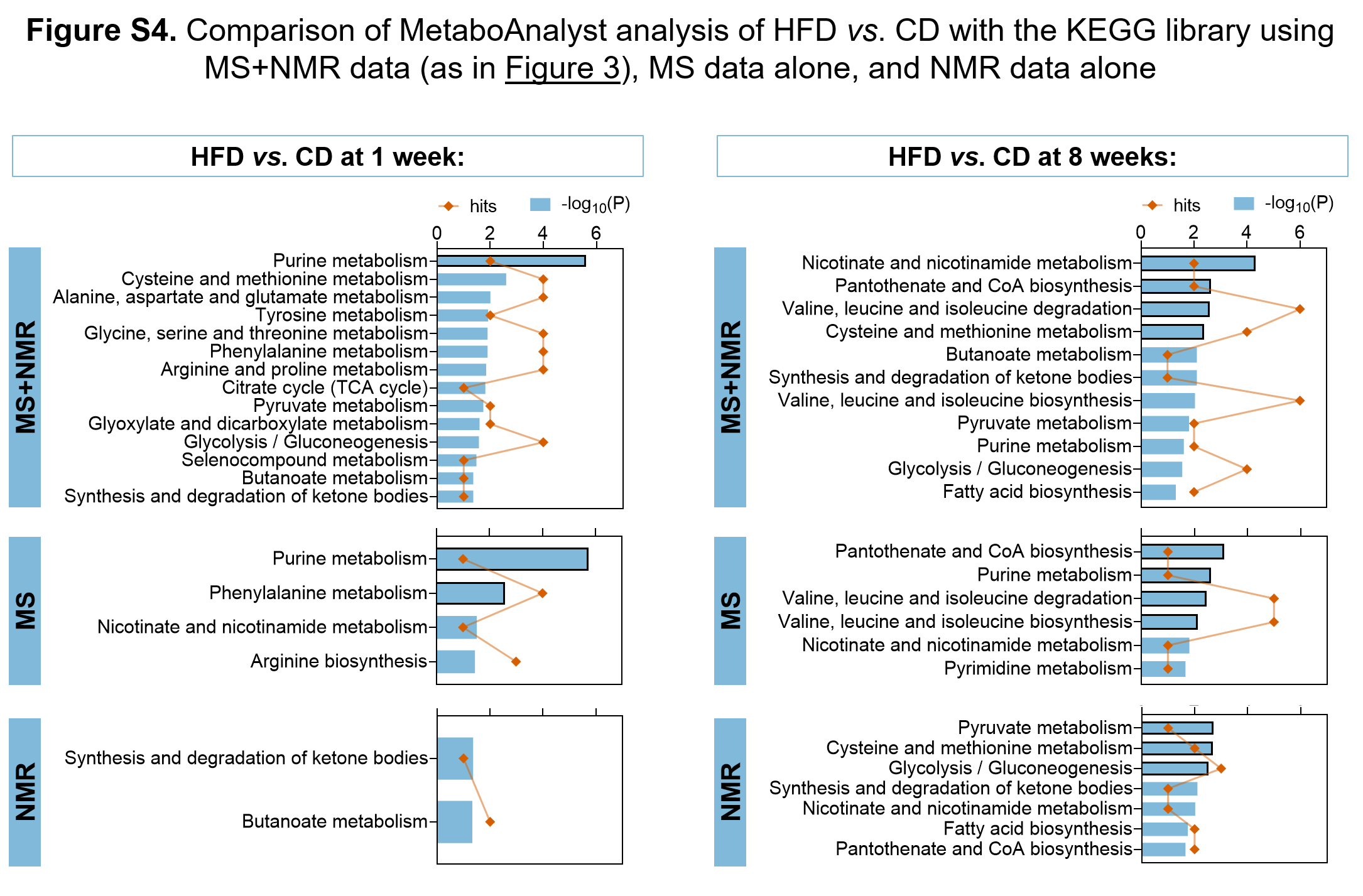

Supplement: Supplementary file 1 [file metabolites-13-00874-s001.zip › Figure S4.tif]

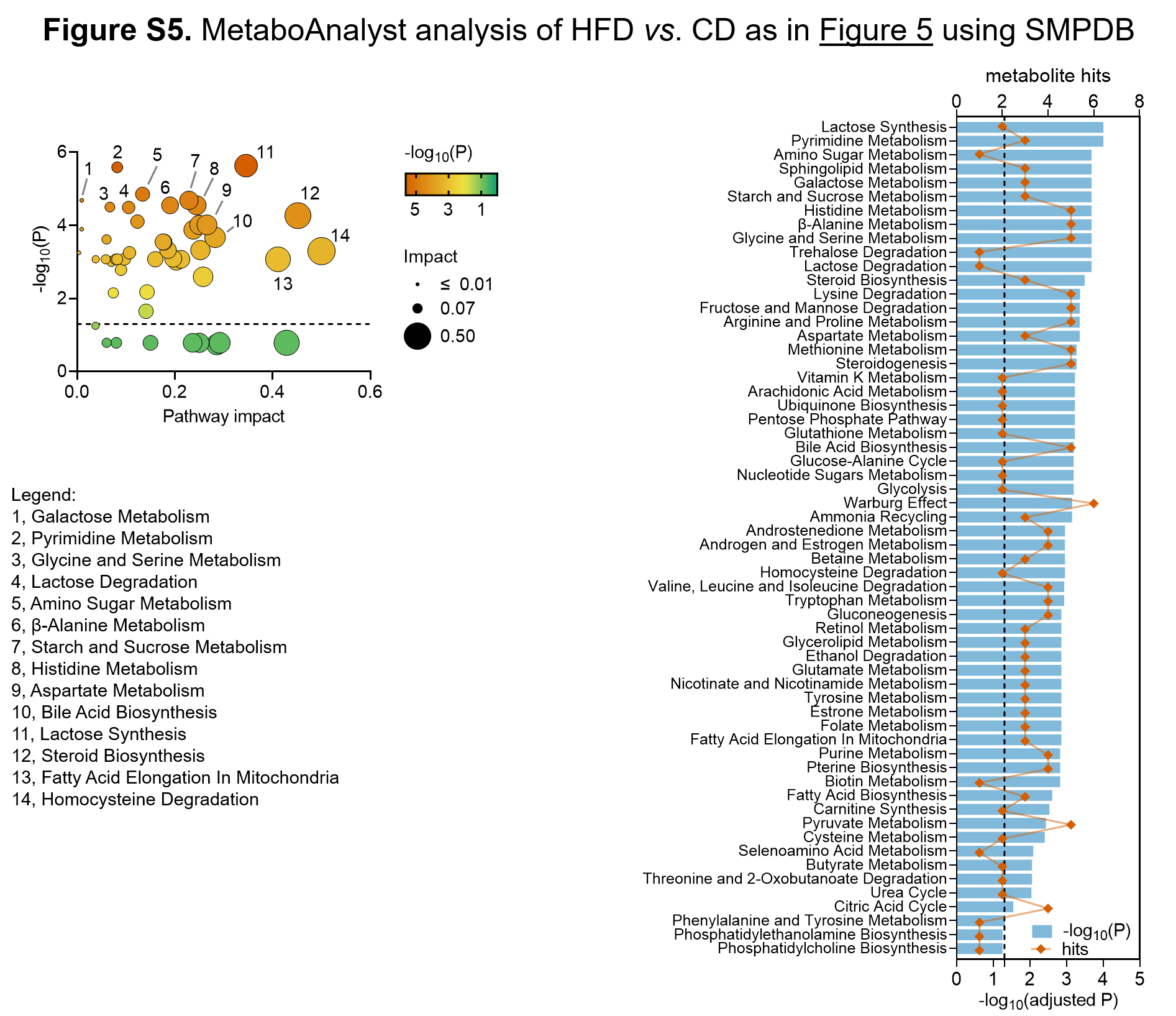

Supplement: Supplementary file 1 [file metabolites-13-00874-s001.zip › Figure S5.tif]

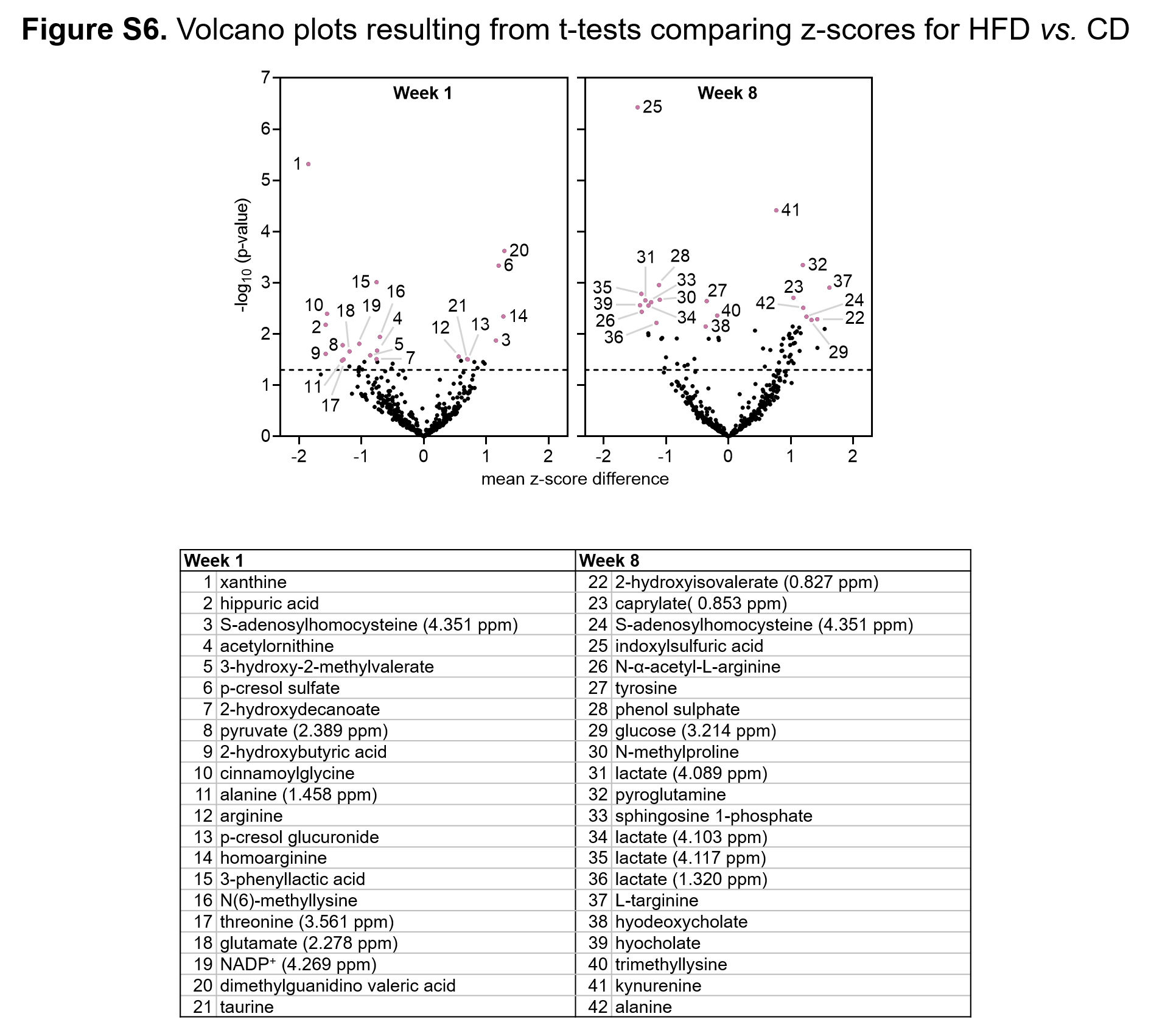

Supplement: Supplementary file 1 [file metabolites-13-00874-s001.zip › Figure S6.tif]
